# Towards targeted screening for acute HIV infections in British Columbia

**DOI:** 10.1186/1758-2652-14-39

**Published:** 2011-08-09

**Authors:** Malcolm Steinberg, Darrel A Cook, Mark Gilbert, Mel Krajden, Devon Haag, Peggy Tsang, Elsie Wong, James I Brooks, Harriet Merks, Michael L Rekart

**Affiliations:** 1 British Columbia Centre for Disease Control, Vancouver, BC, Canada; 2 Faculty of Health Sciences, Simon Fraser University, Burnaby, BC, Canada; 3 Faculty of Medicine, University of British Columbia, Vancouver, BC, Canada; 4 Public Health Agency of Canada, Ottawa, ON, Canada; 5 Faculty of Medicine, University of Ottawa, Ottawa, ON, Canada

## Abstract

**Background:**

Our objective was to describe the characteristics of acute and established HIV infections diagnosed in the Canadian province of British Columbia. Province-wide HIV testing and surveillance data were analyzed to inform recommendations for targeted use of screening algorithms to detect acute HIV infections.

**Methods:**

Acute HIV infection was defined as a confirmed reactive HIV p24 antigen test (or HIV nucleic acid test), a non-reactive or reactive HIV EIA screening test and a non-reactive or indeterminate Western Blot. Characteristics of unique individuals were identified from the British Columbia HIV/AIDS Surveillance System. Primary drug resistance and HIV subtypes were identified by analyzing HIV *pol *sequences from residual sera from newly infected individuals.

**Results:**

From February 2006 to October 2008, 61 individuals met the acute HIV infection case definition, representing 6.2% of the 987 newly diagnosed HIV infections during the analysis period. Acute HIV infection cases were more likely to be men who have sex with men (crude OR 1.71; 95% CI 1.01-2.89], to have had a documented previous negative HIV test result (crude OR 2.89; 95% CI 1.52-5.51), and to have reported a reason for testing due to suspected seroconversion symptoms (crude OR 5.16; 95% CI 2.88-9.23). HIV subtypes and rates of transmitted drug resistance across all classes of drugs were similar in persons with both acute and established HIV infections.

**Conclusions:**

Targeted screening to detect acute HIV infection is a logical public health response to the HIV epidemic. Our findings suggest that acute HIV infection screening strategies, in our setting, are helpful for early diagnosis in men who have sex with men, in persons with seroconversion symptoms and in previously negative repeat testers.

## Background

Acute HIV infection (AHI) has received increasing attention because of its substantial contribution to ongoing HIV transmission [1-6], which has prompted initiatives to identify AHI in order to inform persons of their HIV-positive status during this highly infectious phase. This has been shown to lead to individual behaviour change, which may limit further spread of infection [7]; knowledge of HIV status has been shown to have a marked effect on reducing high-risk sexual behaviour [8].

Current second- and third-generation HIV screening tests have limited ability to detect AHI cases during the seroconversion period. This is also true in settings that utilize rapid or point-of-care HIV antibody testing [9]. Fourth-generation HIV screening tests, which detect both anti-HIV and p24 antigen, have reduced the seroconversion window period [10], and studies have also demonstrated the utility of combining third-generation screening immunoassays with pooled HIV nucleic acid testing (NAT) to shorten the window period between exposure and detection of HIV infection [11]. The North Carolina, US, state programme, which uses pooled NAT for universal screening [12], has not been widely extended to other populations with low HIV prevalence because of high costs. However, there is an emerging consensus supporting the use of AHI screening in settings with high HIV incidence [13,14]. A cut-off of one pooled NAT positive AHI case per 1000 antibody negative samples has been suggested as a guide to select populations to use this approach [15].

The use of pooled NAT has detected between 3.9% and 10.5% additional HIV infections when compared with second- or third-generation antibody screening tests in sexually transmitted infection clinics and sites patronized by men who have sex with men (MSM) [11,16]. However, robust population data to inform criteria for targeted AHI screening using pooled NAT are lacking. In the Canadian province of British Columbia (BC), the majority of HIV tests are conducted in a single laboratory, which enables analysis of provincial data to inform targeted AHI screening. The BC Public Health Microbiology and Reference Laboratory uses third-generation antibody screening, with additional testing for p24 antigen to resolve indeterminate HIV serology, thus enabling a comprehensive review of a series of probable AHIs identified during routine screening.

We compared characteristics of AHIs with all other newly diagnosed HIV infections over a 32-month period to identify criteria to inform enhanced screening strategies for AHI. We also compared HIV subtype distribution and transmitted drug resistance (TDR) patterns between AHI and established HIV-infected individuals over the same time period.

## Methods

Approximately 95% of HIV antibody screening in BC occurs at the Provincial Public Health Microbiology and Reference Laboratory at the BC Centre for Disease Control (BCCDC). Reactive HIV screening tests performed at other BC laboratories and point-of-care sites are referred to the BCCDC, where all HIV confirmatory testing is performed. The current testing algorithm consists of a third-generation enzyme immunoassay (EIA) test (Siemens ADVIA™ Centaur HIV-1/O/2) with confirmation of EIA reactive specimens by HIV-1 Western Blot (WB) (BioRad Genetic Systems™ HIV-1 Western Blot). EIA reactive specimens, with a non-reactive or indeterminate WB, undergo reflex testing for HIV-1 p24 antigen (Biomérieux Vironostika™ HIV-1 Antigen) to help resolve the HIV serological status. p24 antigen reactive specimens are confirmed by a neutralization procedure or by follow-up HIV-1 NAT testing (Roche AMPLICOR™HIV-1 DNA Test, v.1.5).

Province-wide HIV testing and surveillance data from February 2006 to October 2008 were analyzed to inform recommendations for targeted use of AHI screening algorithms for a Canadian Institutes of Health Research-funded study of acute HIV infection. Ethical approval was obtained from the University of British Columbia Clinical Research Ethics Board (UBC-CREB).

The case definition for presumptive AHI included a reactive p24 antigen (confirmed by neutralization or by follow-up HIV-1 NAT) together with a non-reactive or reactive EIA, followed by a non-reactive or indeterminate WB. Presumptive AHI cases needed to meet the public health surveillance case definition for HIV infection, which requires confirmation by follow-up serologic testing or viral load and/or CD4 counts compatible with HIV infection. As the serologic pattern of presumptive AHI cases may be similar to individuals with late-stage HIV infection (i.e., p24 antigen reactive, indeterminate WB), presumptive AHI cases with a reported diagnosis of AIDS within one year were treated as established infections. All other HIV infections diagnosed during the study period were considered to have established HIV infection. All newly diagnosed HIV cases were included in the analysis, i.e., there were no age-based exclusions. Routine demographic information on AHI and established cases was retrieved from the BC HIV/AIDS Surveillance System, which captures past HIV testing history, risk exposure, reason for testing, and geographic location of the test provider. Risk exposure data are not collected from individuals at the time of HIV testing.

Primary drug resistance and HIV subtype determination is routinely performed by the National HIV and Retrovirology Laboratories in Ottawa using residual sera from newly diagnosed HIV infections, as part of the UBC-CREB-approved Canadian HIV Strain and Drug Resistance Surveillance Program. Transmitted drug resistance (TDR) mutations within the protease gene and the first 236 codons of reverse transcriptase, along with subtype, were determined using the Stanford HIVdb Calibrated Population Resistance Tool v.5 beta [http://cpr.stanford.edu/cpr/servlet/CPR], accessed prior to 17 February 2010 [17]. Sequences lacking TDR mutations were classified as wild type.

Demographics, exposure category, HIV testing history, HIV subtype and TDR rates for individuals with AHI and established HIV infection were compared by bivariate analysis in SPSS (v.14.0.2). Independent sample t-tests and Pearson's Chi-square and Fisher's Exact Test were calculated as appropriate. Univariate and multivariable logistic regression analyses were used to identify factors associated with AHI. Variables (gender, MSM, history of prior negative test and presence of AHI symptoms) were chosen in the multivariable model based on statistical significance and clinical relevance. Odds ratios (ORs) and 95% confidence intervals (CIs) were obtained from the logistic regression model, and reported as a measure of association.

## Results

A total of 987 new HIV infections were identified during the 32-month period. Of these, 61 (6.2%) met the case definition for AHI. Three additional cases met the laboratory diagnostic criteria for presumptive AHI, but were not counted as new HIV infections as no confirmatory testing was done, as required by the surveillance case definition. Two other presumptive AHI cases were counted as established infections because they had AIDS case reports within 12 months of diagnosis. Of the 61 confirmed AHI cases, four had a non-reactive third-generation EIA and were tested for HIV-1 p24 antigen on request from the healthcare provider because of symptoms suggestive of a seroconversion illness. All the remaining specimens were third-generation EIA reactive, but the WB on the same specimen was either non-reactive or indeterminate.

Table 1 shows the demographic characteristics, HIV subtype and TDR results for the 61 AHI and 926 established HIV cases. While we did not find a statistically significant difference with respect to sex, the univariate analysis demonstrated that AHI cases had a greater odds of being MSM (crude OR 1.71; 95% CI 1.01-2.89), to have had a documented previous negative HIV test result (crude OR 2.89; 95% CI 1.52-5.51), and to have reported a reason for testing due to seroconversion symptoms (crude OR 5.16; 95% CI 2.88-9.23). When these variables, together with sex (male or female), were included in a logistic regression model, MSM was no longer significantly associated with AHI (Table 2). Reason for testing due to seroconversion symptoms (adjusted OR 4.35; 95% CI 2.39, 7.91) and a documented previous negative HIV test (adjusted OR 2.59; 95% CI 1.35, 4.99) remained significantly associated with AHI.

**Table 1:** Comparison of acute and established HIV infections in British Columbia (N = 987)

Characteristic		Acute cases N = 61	Established cases N = 926	P value	Unadjusted OR
Mean age (Range)		38.6 (20-75 yr)	39.1 (0-81 yr)	0.75	-

Sex	Male	52/61 (85.2%)	735/922 (79.7%)	0.295	1.47 [0.71, 3.04]

Ethnicity	Caucasian	43/60 (71.7%)	540/853 (63.3%)	0.193	1.47 [0.82,2.61]
	
	Aboriginal	9/60 (15.0%)	130/853 (15.2%)	0.960	0.98 [0.47, 2.04]
	
	Other	8/60 (13.3%)	183/853 (21.5%)	0.135	0.56 [0.26, 1.21]

MSM		36/61 (59.0%)	424/926 (45.8%)	0.045	**1.71 [1.01, 2.89]**

IDU		15/61 (24.6%)	266/926 (28.7%)	0.488	0.81 [0.44, 1.47]

Heterosexual		12/61 (19.7%)	195/926 (21.1%)	0.797	0.92 [0.48, 1.76]

Reason for testing due to seroconversion symptoms		20/61 (32.8%)	80/926 (8.6%)	0.000	**5.16 [2.88, 9.23]**

Documented previous negative HIV test		49/61 (80.3%)	542/926 (58.5%)	0.001	**2.89 [1.52, 5.51]**

HIV subtype	A1	0/49 (0.0%)	6/686 (0.9%)	*-*	**-**
	
	B	47/49 (95.9%)	633/686 (92.3%)	0.574	1.97 [0.51, 7.52]
	
	C	0/49 (0.0%)	22/686 (3.2%)	*-*	-
	
	CRF 01_AE	2/49 (4.1%)	11/686 (1.6%)	0.201	2.61 [0.63, 10.87]
	
	Other	0/49 (0.0%)	10/686 (1.5%)	*-*	-

Drug Resistance Class	Wild type	45/49 (91.8%)	611/684 (89.3%)	0.809	1.34 [0.49, 3.69]
	
	NRTI	2/49 (4.1%)	25/684 (3.7%)	0.878	1.12 [0.29, 4.42]
	
	NNRTI	2/49 (4.1%)	28/684 (4.1%)	0.997	1.01 [0.26, 3.91]
	
	PI	1/49 (2.0%)	18/684 (2.6%)	0.802	0.77 [0.13, 4.65]
	
	MDR^a^	1/49 (2.0%)	2/684 (0.3%)	0.064	7.10 [0.92, 55.44]

a. These are included in the totals for each class.

Abbreviations: IDU - injection drug user; MDR - multi-drug resistant; MSM - men who have sex with men; NNRTI - non-nucleoside reverse transcriptase inhibitor; NRTI - nucleoside reverse transcriptase inhibitor; PI - protease inhibitor

**Table 2:** Univariate and multivariable logistic regression analysis for factors associated with acute HIV infection

Characteristic	Unadjusted OR (95% CI)	Adjusted OR (95% CI)
Male sex	1.47 (0.71, 3.04)	1.01 (0.43, 2.37)

MSM	**1.71 (1.01, 2.89)**	1.41 (0.76, 2.63)

Reason for testing due to seroconversion symptoms	**5.16 (2.88, 9.23)**	**4.35 (2.39, 7.91)**

Documented previous negative HIV test	**2.89 (1.52, 5.51)**	**2.59 (1.35, 4.99)**

HIV genotypes and drug resistance mutations were obtained for all new infections for which there was sufficient residual sample volume and from which HIV RNA could be amplified, i.e., 49 of 61 AHIs and 686 of 926 established infections (Table 1). There was no statistically significant difference in the proportions of HIV subtypes between AHIs and established cases. HIV-1 subtype B has accounted for more than 90% of all new HIV infections in BC since the Canadian HIV Strain and Drug Resistance Surveillance Program began in 1998 [18]. Rates of TDR were similar across all classes of drugs for both AHIs and established infections. Multi-drug resistance (MDR), defined as resistance to more than one class of drug, was identified in one case of AHI versus two established cases, resulting in MDR rates of 2% and 0.3%, respectively; however, this was not statistically significant.

## Discussion

AHI detection in this analysis was based on the fact that, during early seroconversion, antibody levels (as reflected by instrument signals) are low and these specimens are typically WB non-reactive or indeterminate and p24 antigen reactive. Using this approach, we were able to confirm AHI in 6.2% of all newly identified HIV cases during the analysis period. There were 57 AHI cases that had a reactive EIA and a non-reactive or indeterminate WB result. In addition, there were four AHI cases identified by p24 antigen testing following a non-reactive screening EIA. These pre-seroconversion cases are identified when providers specifically request p24 antigen testing to help rule out HIV seroconversion syndrome. Systematic pre-seroconversion screening using p24 antigen or pooled NAT testing would likely identify additional such cases.

Given the overall low prevalence of HIV infection among those tested in BC (approximately 0.2%) and the fact that pooled NAT screening of HIV seronegative samples is not performed, the 6.2% yield of AHI is quite high compared with the 3.95% yield from the North Carolina population-based screening programme, which used pooled NAT to test first-generation EIA negative specimens [12].

Two reports have suggested AHI detection thresholds that would support the use of population-based pooled NAT screening. The 0.2% HIV prevalence rate among those tested for HIV in BC is below the 0.55% threshold suggested by Simpson *et al *for the cost-effective use of this strategy [19]. Furthermore, our yield of approximately one AHI per 10,000 specimens screened is less than the one AHI case per 1000 antibody negative samples screened by pooled NAT that has also been suggested as a guideline for implementation of universal pooled NAT screening [15]. Although we did not perform pooled NAT testing on EIA negative specimens, we consider it most unlikely that this approach would have detected 10-fold more AHI cases. While cost-effectiveness studies based on US data may not be directly applicable to HIV screening in Canada, we conclude that a targeted rather than universal pooled NAT screening strategy would be more appropriate for BC [14].

Comparative demographic analysis of the AHI and established HIV cases suggest criteria for targeted AHI screening. First, AHI cases were more likely to occur among repeat testers with a documented previous negative HIV test. Repeat testing may occur more frequently in at-risk individuals who may test soon after risk. This finding could be an underestimate as individuals who previously tested elsewhere, as well as those repeat testers who use inconsistent identifiers, will not have been identified. This suggests that future testing campaigns should encourage repeat testers, especially those at high risk, to sustain regular testing practices and to test following known and/or perceived risk.

Second, a high proportion of AHI cases had a reason for testing due to seroconversion symptoms. This information is sought from healthcare providers or from cases directly during follow up of new positive HIV tests. It is unlikely that all the providers asked for or were informed by their patients about seroconversion symptoms, but these symptoms may be ascertained more frequently by experienced HIV providers working with at-risk populations. Furthermore, at-risk individuals may be more knowledgeable about symptoms of AHI and seek out testing. Thus, this important predictor of AHI is likely under-represented in the AHI cases identified in our analysis. This association of seroconversion symptoms and AHI supports recommendations to educate providers to request specific testing for AHI [20], in addition to routine HIV screening, as soon as symptoms are identified. Educational reinforcement is also required for providers in settings where AHI is frequently observed, as even these providers may fail to consider the diagnosis of AHI [9]. The use of p24 antigen tests to detect AHI among outpatients with fever and other viral symptoms, regardless of HIV risk factors, has been shown to be cost effective [21] and providers working with at-risk populations should adopt a very high index of suspicion with respect to seroconversion symptoms, which are usually non-specific.

Third, while AHI cases were not shown to be significantly more frequent among MSM in the multivariate analysis, 65% of the AHI cases with seroconversion symptoms were MSM. Given that MSM represented 47% of all new HIV infections during the analysis period, we recommend that priority be given to educating MSM and their providers about the importance of seroconversion symptoms as an indicator of AHI. Other studies, including targeted screening in sexually transmitted infection clinics [11], a multi-site study in six US cities [22] and a US Centers for Disease Control study in three public health settings [14], have also documented high proportions of MSM among AHI cases. These and our own findings support recommendations for targeted AHI screening among MSM [14,15], in particular those MSM with seroconversion symptoms.

Since HIV subtyping and TDR testing are routinely performed on new HIV cases, we assessed whether there were differences in HIV subtype distribution and TDR rates between AHI and established cases. If TDR rates are relatively constant over time, we would expect the rates in individuals with AHI to be the same as for established cases [23-25]. TDR rates among our AHI cases are consistent with those reported for acute and recent infections from the United States and Europe [26-31]. In contrast, TDR rates among acute and recent infections in Thailand and Singapore (0% and 1.7%, respectively) are lower than those we observed [32,33]. Among individuals in BC undergoing antiretroviral therapy, the incidence of drug resistance has steadily declined since 1996 [34]. No statistically significant differences in subtype or TDR rates, including MDR, were observed. However, given the small AHI sample size, our analysis may have been underpowered.

Our data analysis reflects a convenience sample of individuals who chose to undergo HIV testing or were referred for testing by their healthcare provider, and p24 testing of EIA negative individuals only occurred when specifically requested. This limits the generalizability of our findings in determining the value of AHI screening as a health policy. Other limitations include the limited capacity of the provincial surveillance database to describe features of individuals with a new positive HIV test. Geographic areas where AHI cases are concentrated has been shown by others to be useful to identify sites for targeted AHI screening [13], but we did not analyze this variable since the surveillance database captures only the location of the healthcare provider who ordered the test.

In addition, we were not able to link co-sexually transmitted infection (STI) status and/or history of previous STIs to AHI cases. Previous studies have shown high yields of AHI with targeted screening in STI clinics and we expect that our AHI cases would also be more likely to have a history of STIs and/or a current STI at the time of HIV diagnosis. Shepard *et al *emphasize that the majority of AHI cases detected in their STI clinic study were found at one STI clinic, of which approximately one-third of patients identified as MSM [35]. This supports our consideration to prioritize STI clinic sites that reach out to MSM and other high-risk populations for targeted AHI screening.

We cannot rule out the possibility that some of our AHI cases represent late-stage disease, at which time p24 antigen may be detectable and waning antibodies may result in an indeterminate WB. Two presumptive AHI cases were classified as established infections because AIDS was reported within 12 months of HIV diagnosis. However, delays in and incomplete AIDS case reporting may have resulted in an overestimation of the number of AHI cases as a result of misclassification of late-stage disease.

## Conclusions

There have been calls for more rigorous cost-effectiveness studies to inform AHI screening strategies [11,14]. We believe that our study provides insights for a targeted screening strategy to enhance detection of AHI in BC. Our results confirm that we should continue to investigate EIA reactive, and WB indeterminate or non-reactive, results and possibly switch from p24 antigen testing to the more sensitive NAT to detect AHI. Further, we should target testing sites reaching out to MSM and consider testing with an optimally designed pooled NAT protocol [36]. Our findings support our collaboration with community-based initiatives in BC to promote awareness of AHI and offer enhanced AHI screening to MSM [37]. This includes developing risk profile protocols for referring persons with non-reactive point-of-care HIV test results to undergo pooled NAT testing.

Our results also encourage us to increase and sustain educational initiatives for healthcare providers requesting HIV tests. The key messages are to promote a high index of suspicion about seroconversion illness symptoms, especially in high-risk patients, and to act on this through requests for AHI screening, and to inquire about previous HIV testing to inform similar requests for AHI screening.

Every HIV infection identified beyond the AHI stage is a missed opportunity to promote risk reduction when transmission dynamics are favourable for onward transmission. Targeted screening initiatives to enhance detection of AHI are a logical public health response to the HIV epidemic. Prevention programmes for persons with newly diagnosed HIV infection need to be ready to respond to individuals with AHI once identified [11].

## Competing interests

The authors declare that they have no competing interests.

## Authors' contributions

MS, DAC, MG, MK and MLR conceived and designed the study. MS and DAC drafted the manuscript. DH, EW and MG extracted and analyzed the case surveillance data. PT performed the HIV screen testing and confirmation. HM and JIB performed the TDR testing and HIV subtyping and analyzed the data. All authors provided critical review of the manuscript and approved the final version to be published.
